# A comparison between spinal cord infarction and neuromyelitis optica spectrum disorders: Clinical and MRI studies

**DOI:** 10.1038/s41598-019-43606-8

**Published:** 2019-05-15

**Authors:** Jung Lung Hsu, Mei-Yun Cheng, Ming-Feng Liao, Hui-Ching Hsu, Yi-Ching Weng, Kuo-Hsuan Chang, Hong-Shiu Chang, Hung-Chou Kuo, Chin-Chang Huang, Rong-Kuo Lyu, Kun-Ju Lin, Long-Sun Ro

**Affiliations:** 1 Department of Neurology, Chang Gung Memorial Hospital Linkou Medical Center and College of Medicine, Chang-Gung University, Linkou, Taoyuan, Taiwan; 2 Graduate Institute of Mind, Brain, and Consciousness, Taipei Medical University, Taipei and Brain and Consciousness Research Center, TMU Shuang Ho Hospital, New Taipei City, Taiwan; 3 Institute of Molecular Medicine, National Tsing Hua University, Hsinchu, Taiwan; 4 Department of Traditional Chinese Medicine, Division of Chinese Acupuncture and Traumatology, Chang Gung Memorial Hospital, Linkou Medical Center and Chang Gung University College of Medicine, Taipei, Taiwan; 5 Department of Nuclear Medicine and Center for Advanced Molecular Imaging and Translation, Linkou Chang Gung Memorial Hospital, Taoyuan, Taiwan; 6 Department of Medical Imaging and Radiological Sciences and Healthy Aging Research Center, Chang Gung University, Taoyuan, Taiwan

**Keywords:** Neurology, Demyelinating diseases

## Abstract

This study aims to investigate the clinical features and magnetic resonance imaging (MRI) findings in patients with spinal cord infarction (SCI) and neuromyelitis optica spectrum disorders (NMOSDs). Over a period of 16 years, we retrospectively analyzed 39 patients with SCI and 21 patients with NMOSD. The demographic features and clinical presentations of both diseases were carefully documented. Etiology-specific MRI features, such as the length and distribution of the lesions, the owl’s eyes sign and bright spotty lesions, were recorded and analyzed regarding their association with the clinical signs/symptoms. Patients with SCI were older than patients with NMOSD and had sudden onset of clinical symptoms with focal pain adjacent to the lesions. Concomitant spinal cord and vertebral body infarctions were frequently associated with aortic pathology (p = 0.04). In addition, artery dissection was highly associated with combined ASA and unilateral PSA infarctions and long segments of SCI (all p < 0.05). In contrast, patients with NMOSD had a relatively younger age of onset, female predominance and subacute progression of limbs weakness. As observed by MRI, the length and location of the lesions demonstrated significant differences between the two diseases (P < 0.01). The owl’s eyes sign showed more frequently in patients with SCI than NMOSD (p < 0.01). The predicted prognoses in SCI and NMOSD were significantly associated with initial motor function (muscle power), after adjustments for age and gender (p < 0.01 and p = 0.02, respectively). Along with patient demographic characteristics, lesion features on MRI can help clinicians differentiate acute noncompressive myelopathy due to SCI from that due to NMOSD, which may lead to immediate initiation of adequate therapeutic measures.

## Introduction

Acute noncompressive myelopathy is a neurological emergency characterized by a rapid progression of sensorimotor deficits with or without sphincter disturbances. The diagnosis of acute noncompressive myelopathy presents a challenge to clinicians, as several possible etiologies, such as vasculature, demyelination and inflammation, need to be considered in the differential diagnoses^1–3^. In acute noncompressive myelopathy, spinal cord infarction (SCI) could mimic neuromyelitis optica spectrum disorders (NMOSD) because of several overlaps in the clinical presentations and magnetic resonance imaging (MRI) findings^4,5^. However, the treatment and prognosis may be quite different in the two diseases. Therefore, a correct diagnosis of each disease, which can prevent delays in adequate therapeutic measures or subsequent unwarranted, potentially harmful immunosuppressive therapies, is mandatory^6,7^.

SCI is a rare disease that represents only approximately 1% of all strokes, but it may leave the patient with devastating neurological sequels, such as paraplegia or quadriplegia^8,9^. In Taiwan, NMOSD is also a rare condition, and it has a significantly higher proportion of middle-aged female than male patients, exhibits a high rate of relapses and results in greater disability than conventional multiple sclerosis^10,11^. It would be advantageous to know whether clinical and MRI parameters could distinguish between these two diseases^12^. In previous studies, several etiology-specific MRI features have been proposed to differentiate SCI from NMOSD, such as the location, length extension and intensity texture of cross-sections of the lesion in the spinal cord^12^. To help clinicians gain a better awareness of both diseases, we performed a retrospective study to evaluate the detailed clinical history, spinal cord MRI features and cerebrospinal fluid (CSF) characteristics at our institution (a tertiary medical center) in a group of patients with SCI or NMOSD.

## Materials and Methods

### Study design and patient population

We retrospectively analyzed the clinical presentations, initial neurological examinations, MRI features and CSF profiles of a total of 66 patients with SCI or NMOSD from 2002 to 2018. The study protocol was approved by the institutional review board of the Chang Gung Memorial Hospital (IRB number: 201800769B0). All methods were performed in accordance with the relevant guidelines and regulations. Six patients were excluded from this study due to a lack of complete clinical information or imaging data, and 60 patients in total were included in the analyses. We recorded demographic characteristics, medical histories, and information on the clinical presentation, including the temporal profiles, initial symptoms, presence of acute focal pain adjacent to the spinal cord lesion and neurologic examinations. The CSF studies and MRI features, including the topography of the lesions at the initial assessment, were analyzed. The diagnosis of SCI was defined as acute myelopathy with hyperintense lesions in a defined vascular territory on T2-weighted images. Stroke risk factors or mechanisms that could account for the clinical presentations were identified, and other etiologies were excluded^13^. The diagnosis of NMOSD was based on the international consensus criteria for NMOSD^14^.

The temporal profiles from the initial symptoms onset to the nadir of neurologic dysfunction were classified as hyperacute (<6 hours), acute (6–48 hours) and subacute (>48 hours) patterns^13^. The nadir was defined as the point of the worst neurologic function, before improvement or plateau, based on history and neurologic examinations. For each limb, a Medical Research Council (MRC) score (from 0 to 5) was assigned to represent the muscle power in that limb. The muscle power values in the upper limbs and lower limbs were measured by averaging the MRC scores from both sides. The total muscle power in all four limbs was calculated by summating the MRC scores of all the limbs. Associated medial history information, such as vascular risk factors (hypertension, diabetes mellitus and dyslipidemia), previous trauma and prior fever or upper respiratory tract infections, was also recorded. CSF profiles, including pleocytosis, sugar, protein, immunoglobulin G index, and oligoclonal band (OCB) measurements, were documented. Serum anti-aquaporin-4 (AQP4) antibody testing was performed by enzyme-linked immunosorbent assay (ELISA)^15^. We used modified Rankin scale (mRS) scores to compare the one-month outcomes between the SCI patients and the NMOSD patients^16^.

### Evaluation of MRI parameters

MRI of the spine obtained within two weeks of admission was reviewed by a board-certified neuroradiologist who was blinded to the clinical diagnosis. All studies included axial and sagittal T1- and T2-weighted sequences that imaged the spine. The length of each lesion was measured by the sagittal extension of T2 hyperintense areas using the number of vertebral body spans. The location of each lesion was recorded according to the vertebral body level (e.g., cervical, thoracic or lumbar). Adjacent vertebral body infarction, defined by the geographic marrow hyperintensity on the sagittal T2-weighted images, was also evaluated^17,18^. Gadolinium enhancement [Gd+] on T1-weighted images was recorded as present or absent. The midpoint of each lesion was characterized using the axial T2-weighted images. The distribution of lesions in cross-sections of the spinal cord was classified according to the anterior, posterior, lateral and central regions^13^. In addition, the vascular territory of the spinal cord was used to fit for the territory involved in the artery infarction (anterior spinal artery (ASA) and posterior spinal artery (PSA)) in patients with SCI^19^. The “owl’s eyes sign” feature, defined as the “bilateral hyperintensities of the anterior horns on axial T2-weighted images” seen in SCI, and “bright spotty lesions”, defined as the “very hyperintense spotty lesions on axial T2-weighted images that are visually more hyperintense than those of surrounding CSF without flow void effects” seen in NMOSD, were recorded as present or absent in all patients^20,21^.

### Statistical analyses

All statistical analyses were performed using SPSS (version 21.0). Continuous variables are expressed as the means ± standard deviations. Categorical variables are presented as numbers and ratios. Independent t-tests were performed to compare the mean age between the groups. Chi-square tests, Fisher’s exact tests and Mann-Whitney U tests were used to compare patients with SCI and NMOSD in terms of gender, clinical presentation, one-month outcomes and imaging characteristics. Logistic regression analysis was used to study the association between clinical symptoms, short-term outcomes and MRI characteristics after adjustments for age and gender effects. Statistical significance was defined as p < 0.05.

## Results

**Figure 1:**
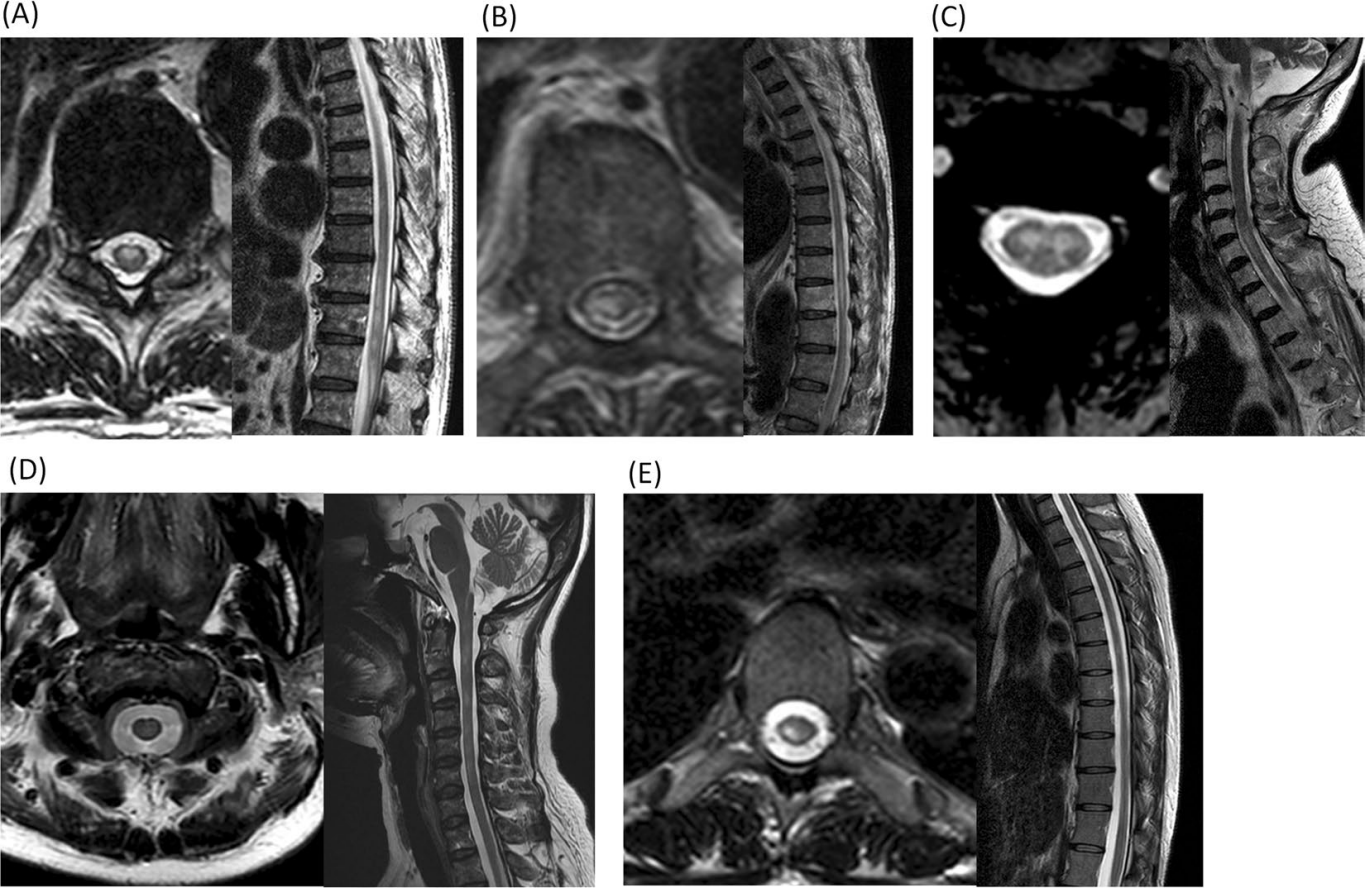
Typical MRI scans of acute myelopathy due to SCI and NMOSD. (**A**) An 83-year-old man had an infrarenal abdominal aorta aneurysm. After operation, he suffered from acute paraparesis with urine incontinence. His spine MRI scans showed a typical ASA infarction (axial view) at the T10–T12 levels (sagittal view). (**B**) A 79-year-old man had a history of abdominal aortic aneurysm dissection. After operation, he had acute paraplegia with urine incontinence. His spine MRI scans showed combined ASA and right PSA infarctions (axial view) at the T8–T10 levels (sagittal view). (**C**) A 57-year-old woman had a sudden onset of quadriparesis with neck pain and urine incontinence. The MRI scans showed a typical owl’s eyes sign (axial view) at the C5–7 levels (sagittal view). (**D**) A 49-year-old female had progressive paraparesis for 7 days. Her spine MRI scans showed transverse myelopathy (axial view) at the C2–C6 levels (sagittal view). (**E**) A 45-year-old female had a subacute onset of progressive paraparesis. Her MRI scans showed typical bright spotty lesions (axial view) and a hyperintense lesion at the T2–T4 levels (sagittal view).

Figure 1 demonstrates the typical imaging patterns obtained from subjects presenting with acute myelopathy due to SCI or NMOSD. During the study period, 39 patients had a diagnosis of SCI, and 21 had a diagnosis of NMOSD. Table 1 presents a summary of the differences between the two groups. In patients with SCI, our 39 subjects (22 male, 17 female) had a mean onset age of 57.4 ± 18.5 years (range = 18–87 years), with the mean age being 56.5 years in men and 58.4 years in women (p = 0.76, independent two-sample t-test). In the 21 patients with NMOSD, the mean onset age was younger than that of patients with SCI and showed a female predominance (all p < 0.05). Among the vascular risk factors, a history of hypertension more frequently occurred in patients with SCI than in those with NMOSD (p < 0.05). Nine patients with SCI had aortic dissections or had histories of aortic operations; of these, the most commonly involved vertebral body segments were the T10–T12 regions. Based on the classification of the vascular territory, 35 patients had SCI attributed to ASA infarctions, and 4 patients had a combination of ASA with unilateral PSA infarctions.

**Table 1:** Comparisons of the clinical features between patients with SCI and patients with NMOSD.

	SCI (N = 39)	NMOSD (N = 21)	p value
Onset age	57.4 ± 18.5	42.0 ± 12.6	<0.01
Gender (M:F)	22:17	0:21	<0.01
Hypertension (Y:N)	20:19	1:20	<0.01
Diabetes mellitus (Y:N)	10:29	2:19	0.12
Dyslipidemia (Y:N)	12:27	2:19	0.05
Fever (Y:N)	3:36	3:18	0.81
Temporal profile of onset: *Hyperacute* (<6 *hours*)	39	0	<0.01
*Acute* (*6–*48 *hours*)	0	2	<0.01
*Subacute* (>*48* *hours*)	0	19	<0.01
Onset to nadir time	10.7 ± 13.1 (minutes)	8.1 ± 6.1 (days)	<0.01
Focal pain adjacent to lesion (Y:N)	19:20	1:20	<0.01
All limbs muscle power (MRC score)	11.7 ± 4.5	15.9 ± 3.3	<0.01
Upper limbs muscle power (MRC score)	4.2 ± 1.4	4.5 ± 0.7	0.29
Lower limbs muscle power (MRC score)	1.5 ± 1.7	3.3 ± 1.5	<0.01
Hyporeflexia in affected limbs (Y:N)	17:22	1:20	<0.01
Sphincter incontinence (Y:N)	29:10	7:13	<0.01
mRS score (one month later)	3.8 ± 1.2	2.4 ± 1.2	<0.01

SCI: spinal cord infarction; NMOSD: neuromyelitis optica spectrum disorders; MRC: Medical Research Council; mRS: modified Rankin scale; Y: yes; N: no.

### Clinical findings

The temporal profiles of onset symptoms showed significant differences between the patients with SCI and the patients with NMOSD. Patients with SCI more frequently presented with hyperacute onset than those with NMOSD (p < 0.05). The mean onset time to nadir was approximately 10 minutes in patients with SCI and 8 days in patients with NMOSD (p < 0.05). Acute focal pain adjacent to the lesion level more frequently occurred in SCI (49%) than in NMOSD (5%) patients (p < 0.05). One patient with NMOSD had focal neck pain with left hand numbness before the onset of weakness. The pain subsided after steroid treatment (Table 1). Regarding the neurological examinations, patients with SCI had lower MRC scores in all/lower limbs and more commonly had hyporeflexia in the affected limbs than patients with NMOSD (p < 0.05). Sphincter disturbances were more frequent in patients with SCI than in those with NMOSD (p < 0.05). In patients with SCI, 28 out of the 29 patients had urine retention symptoms.

Serum levels of anti-AQP4 antibody were studied in two patients with SCI and 20 patients with NMOSD. There were significantly higher levels of anti-AQP4 antibody in patients with NMOSD than in patients with SCI (NMOSD = 108.0 ± 93.9; SCI = 1.3 ± 0.3 unit/mL, p < 0.01. The reference value was <3 unit/mL). In patients with NMOSD, the levels of anti-AQP4 antibody did not show any significant associations with age, MRC scores of all limbs, lesion lengths or one-month outcomes (p = 0.53, 0.29, 0.81 and 0.42, respectively).

Eleven patients with SCI and 12 patients with NMOSD underwent CSF studies during the admission period. There were no significant group differences in sugar levels, total protein levels or pleocytosis (Table 2). In patients with SCI, 10 of 11 patients had lymphocyte counts below 5 cells/uL and one patient had a traumatic tapping was excluded for study. Four patients with SCI and seven patients with NMOSD underwent immunoglobulin G index studies. The results did not show any significant differences (p = 0.06). In the OCB study, seven patients with SCI and eight patients with NMOSD were evaluated. None had positive results.

**Table 2:** Comparisons of the CSF features between patients with SCI and patients with NMOSD.

	SCI (N = 10)	NMOSD (N = 12)	p value
Protein (mg/dL)	51.7 ± 32.2	45.7 ± 11.3	0.57
Sugar (mg/dL)	80.0 ± 17.4	66.7 ± 15.9	0.08
Cell (RBC)	0.8 ± 1.4	8.7 ± 11.7	0.06
Cell (lymphocyte)	0.8 ± 1.3	9.0 ± 20.3	0.25
Immunoglobulin G index	0.69 ± 0.08 (N = 4)	0.57 ± 0.06 (N = 7)	0.06
Oligoclonal bands (OCB)	Negative (N = 7)	Negative (N = 8)	

SCI: spinal cord infarction; NMOSD: neuromyelitis optica spectrum disorders.

Regarding short-term outcomes, patients with SCI had significantly higher mRS scores than those with NMOSD, which indicated a poor prognosis in patients with SCI. To study the factors that contribute to short-term outcomes, a regression analysis was performed. Our results showed that the initial total MRC scores had a significant association with short-term outcomes at one month, after adjusting for age and gender, in patients with SCI and those with NMOSD (p < 0.01 and p = 0.02, respectively).

### MRI characteristics

**Figure 2:**
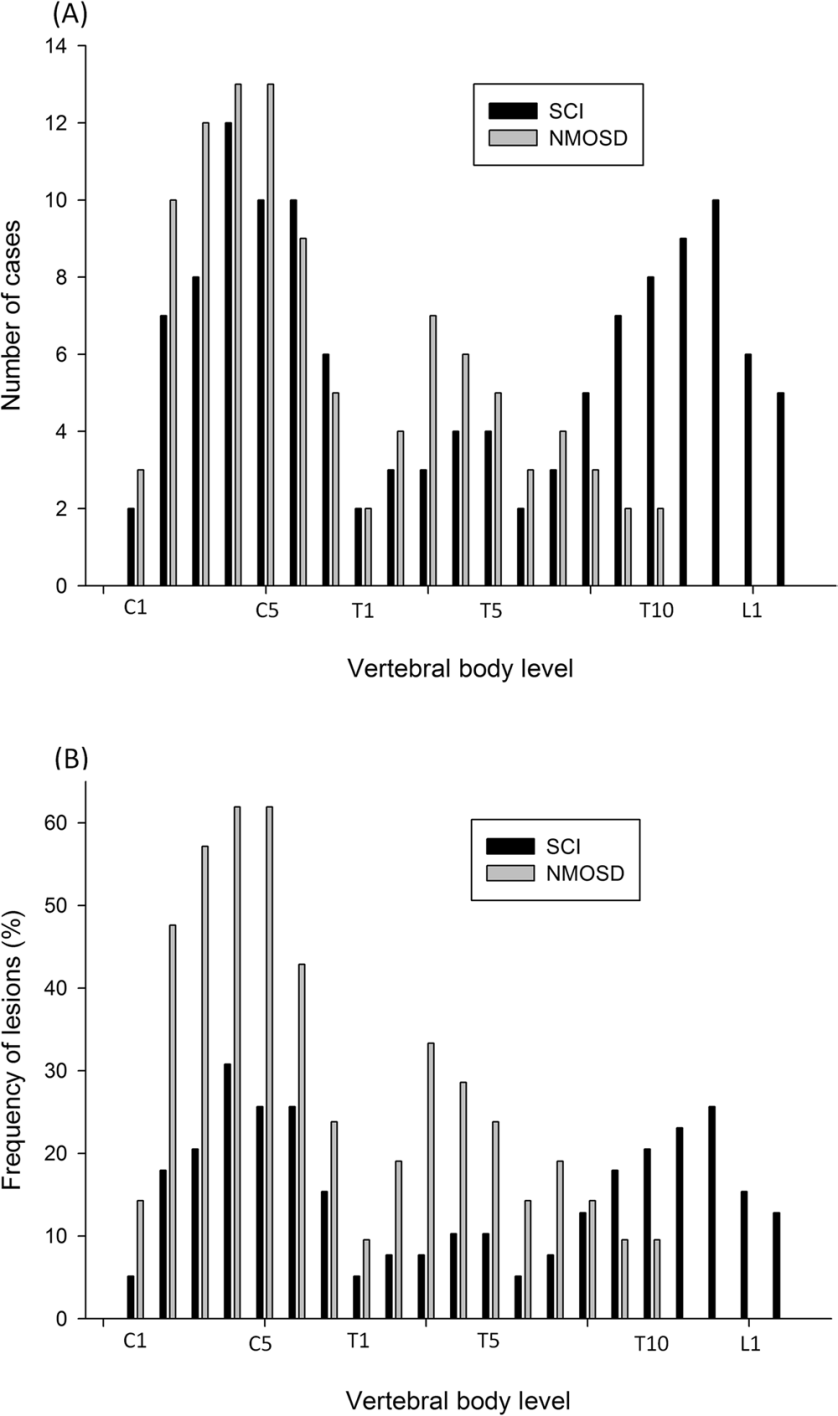
Histograms demonstrating the distributions of the (**A**) number of cases and (**B**) frequencies of the lesions along the vertebral body levels among patients with SCI and patients with NMOSD.

Figure 2 demonstrates the number of cases and the ratios of lesions at different vertebral body levels in patients with SCI and those with NMOSD. Patients with NMOSD frequently had lesions in the cervical and upper thoracic vertebral body levels. On the other hand, patients with SCI frequently had lesions in the lower thoracic and lumbar levels. Table 3 presents a summary of the MRI differences between the two groups. Contrast enhancements on the T1-weighted MRI were more commonly seen in patients with NMOSD than in patients with SCI during acute presentations. In patients with NMOSD, 62% of the patients had contrast enhancements. In contrast, only two patients with SCI had contrast enhancements on the T1-weighted MRI. The lesion length (mean of the vertebral body span) was significantly longer in patients with NMOSD than in patients with SCI (NMOSD = 5.0 ± 1.7; SCI = 3.1 ± 1.5, p < 0.01). We divided the length of the lesion into long segments (≥3 vertebral body spans) and short segments (<3 vertebral body spans) then performed Fisher’s exact test between the lengths of lesions and vessel dissection in patients with SCI. In 22 patients with long segments of lesions, 11 patients had vessel dissections (either aortic or vertebral artery dissections). The results showed a significant association between the lengths of the lesions and vessel dissections (p = 0.0009).

**Table 3:** Comparisons of the MRI features between patients with SCI and patients with NMOSD.

	SCI (N = 39)	NMOSD (N = 21)	p value
Contrast enhancement (Y:N)	2:37	13:7	<0.01
Lesion length (vertebral body span)	3.1 ± 1.5	5.0 ± 1.7	<0.01
Vertebral body infarction (Y:N)	8:31	0:21	<0.01
Owl’s eyes sign (Y:N)	11:28	0:21	<0.01
Bright spotty lesions (Y:N)	1:31	14:7	<0.01
Axial anterior pattern	28:9	3:18	<0.01
Axial central pattern	24:13	12:9	0.56
Axial lateral pattern	27:10	11:10	0.11
Axial posterior pattern	9:28	9:12	0.14

SCI: spinal cord infarction; NMOSD: neuromyelitis optica spectrum disorders; Y: yes; N: no.

Regarding vertebral body infarction, eight patients with SCI had this feature, which was a significantly higher proportion (20%) than that in patients with NMOSD (0%) (p < 0.01). We further studied the vertebral body infarction sign in patients with or without aortic pathology, and our results showed a significantly higher proportion of patients with aortic pathology than those without (Chi-square test, p = 0.04). As observed on the axial MR images, the owl’s eyes sign was more common in patients with SCI (Chi-square test, p < 0.01); in contrast, bright spotty lesions were more frequently seen in patients with NMOSD (p < 0.01). In the current study, the owl’s eyes sign was present with the ASA infarctions (31%) but not with the combination of ASA and unilateral PSA infarctions (0%). The pattern of lesion distributions in the axial view more frequently affected the anterior portion in patients with SCI than in those with NMOSD (p < 0.01).

After logistic regression analyses, the MRC scores of all limbs did not show significant associations with lesion length or the pattern of axial distribution of lesions (p = 0.39 and 0.85, respectively). The MRC scores of the all limbs showed no associated with the owl’s eyes sign (p = 0.08), even after adjusting for age and gender, in patients with SCI. On the other hand, the bright spotty lesions were also not associated with MRC scores in patients with NMOSD (p = 0.56). The lesion length, lesion pattern, owl’s eyes sign and bright spotty lesions did not show significant associations with short-term outcomes, even after adjustments for the effect of age and gender, in patients with SCI and patients with NMOSD (p = 0.08, 0.47, 0.23 and 0.58, respectively).

## Discussion

In our study, patients with NMOSD had younger onset ages, a female predominance, and a subacute onset of clinical presentations, higher MRC scores and better one-month outcomes than patients with SCI. In contrast, patients with SCI had older onset ages, multiple vascular risk factors, and more frequent sudden onset of clinical presentations than patients with NMOSD. In patients with SCI, the mean time interval from initial symptom onset to the nadir was approximately 10 minutes, which was similar to that observed in a previous report^22^. Focal pain adjacent to the spinal cord lesion was more common in these patients. Regarding one-month outcomes, patients with NMOSD had lower mRS scores, which indicated better outcomes were achieved in these patients than in those with SCI. This result was consistent with those of a previous study^23^. In patients with SCI, poor initial muscle power (MRC scores) was associated with poor short-term outcomes, a finding that aligned with those of previous studies, in which severe initial motor impairment was an independent predictor of unfavorable outcomes^9,22^.

### Spinal cord vasculature contributes to MRI features in patients with NMOSD and SCI

**Figure 3:**
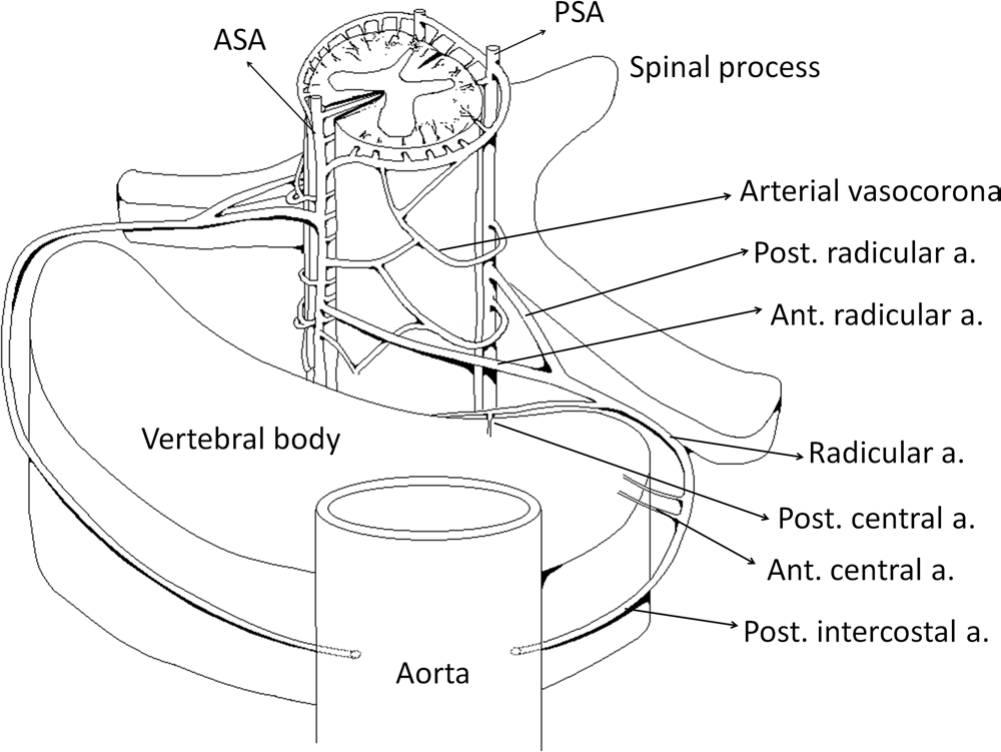
A three-dimensional diagram of the arteries of the spinal cord vascular anatomy illustrates the arrangement of the cord, vertebra and major vessels. The posterior intercostal artery branches off the anterior and posterior radicular arteries. The arterial vasocorona connects the ASA and PSA.

The spinal cord has a highly complex and variable vascular anatomy among different individuals. Along the rostral to caudal axis, the vascular area is mainly supplied by one ASA and two PSAs. All three arteries branch off from the vertebral arteries, and the ASA supplies the anterior two-thirds of the cord, while the PSA pairs supply the rest. These three arteries finally anastomose at the conus medullaris, where the spinal cord ends, usually at the level of the L1 or L2 vertebra^19^. Figure 3 is a simplified diagram that illustrates the arrangement of the cord, vertebra and major vessels^24,25^. The radicular arteries originate in the vertebral artery or posterior intercostal arteries of the aorta, enter the spinal foramen, and then branches off the anterior and posterior radicular arteries, which anastomose to the ASA and PSA, respectively. The arterial vasocorona supplies the underlying spinal cord and connects to the ASA and PSA^24^. In the thoracic-lumbar region, the largest radicular artery is called the artery of Adamkiewicz, which originates from the aorta and mainly supplies these locations. The spinal cord in these areas is particularly dependent on this artery, making it the most vulnerable region in acute aortic pathologies^9^.

The MRI features of patients with SCI included more frequent lesions in the lower thoracic and lumbar levels than were found in patients with NMOSD. The lesions were characterized by shorter lengths and anterior patterns in the axial view. However, neither lesion length nor the distribution pattern of lesions in the axial view was significantly associated with short-term outcomes in patients with SCI. The prevalence of the owl’s eye sign was 28% in patients with SCI in the current study. In previous studies, the prevalence of the owl’s eye sign ranged from 5% to 80%^26,27^. A previous study showed that the owl’s eye sign could not be used to differentiate patients with SCI from those with NMOSD. Our study shows that this sign is associated with patients with SCI but not those with NMOSD^12^. The lower frequency of owl’s eye observed in NMOSD may be related to the fact that longitudinally extensive transverse myelitis lesions are one of the core clinical characteristics in the NMOSD criteria^14^.

The vertebral body infarction sign, indicated by an abnormal bone marrow signal on T2-weighted imaging, was predominantly observed in the anterior half or in multiple areas near the endplate and/or deep medullary portion of the vertebral body^28^. In the current study, the prevalence of the vertebral body infarction sign was 20%, which was similar to that observed in our previous study^27^. In previous reviews, this sign was reported in approximately 14–35% of patients and could appear as early as eight hours after the onset of symptoms or as late as a few days later^17,28^. Faig J *et al*. showed that aortic pathology was frequently observed with vertebral body infarctions^17^. In the current study, nine cases with aortic diseases were included, and 44% of these patients had this sign. A Chi-square test showed that these aortic pathology was significantly more associated than no aortic pathology with the vertebral body infarction sign (p = 0.04). This result was similar to that achieved in our previous study showing that concomitant SCI and vertebral body infarction signs are highly associated with aortic pathology^27^. However, this sign is not specific for SCI and can also be seen in other aetiologies, such as infection, fracture or metastasis^17^. A complete clinical history and the exclusion of other possibilities are key to achieving a correct diagnosis.

However, patients with NMOSD typically present with involvement of the cervicothoracic levels, which contain a junction area with abundant collateral circulation and are less likely to be involved in SCI^12^. In our study, approximately 50–62% of patients with NMOSD had lesions distributed within the C2–C5 regions. Patients with NMOSD had longer lesion lengths and more frequent contrast enhancements in the lesions than were found in patients with SCI. In our study, bright spotty lesions were frequently present (66%), and 65% of the patients showed contrast enhancements. These results were consistent with those of a previous report, in which 86% of NMOSD patients had bright spotty lesions, and 65% had contrast enhancements^20^.

### Clinical features of SCI

Patients with SCI usually reported focal pain as one of the initial symptoms, at a frequency ranging from 59% to 73%^9,19^. In the current study, 49% of the patients had focal adjacent pain at the initial onset of symptoms. The mechanism of pain may be related to ischemia of the local meninges, the vertebral body or the nerve root^29,30^. The pathogenesis of SCI may be related to global hypoperfusion or the occlusion of the radicular artery^19,31^. Cardiac/aortic surgery, intervertebral disk compression or spine trauma have also been reported^32^. We studied associations between 35 patients with ASA infarctions and four patients with both ASA and unilateral PSA infarctions and vessel dissections. Interestingly, according to the logistic regression, we found that vessel dissections (e.g., vertebral artery dissection or aortic dissection) were more frequently associated with patients with both ASA and unilateral PSA than patients with ASA infarctions (p < 0.05, R-square = 0.28). In addition, patients with vessel dissections also demonstrated a significant association with lesion length according to a Chi-square test (p < 0.05). We suggest that artery dissection may block the orifice of the intercostal artery and subsequently occlude the radicular artery, leading to a combination of ASA and unilateral PSA infarctions (see Fig. 3). The lengths of the lesions were longer in patients with both ASA and unilateral PSA infarctions than in those without. However, future large and prospective studies are needed to confirm this observation.

### Clinical features in patients with NMOSD

NMOSD is an inflammatory disease mainly characterized by optic neuritis and long, extended spinal cord lesions^14^. NMOSD frequently displays a relapsing-remitting course, and approximately 60–90% of patients have anti-AQP4 antibodies^33,34^. In the current study, the serum anti-AQP4 antibody-positive rate was 86%, similar to the rate observed in a previous study^35^. In previous studies, the prognostic factors identified in patients with NMOSD were onset age, ethnic group and anti-AQP4 antibody levels^36,37^. In our results, the onset age and anti-AQP4 antibody levels were not significantly associated with mRS scores (p = 0.19 and p = 0.47, respectively). These associations may have resulted from our small sample size and the fact that prognoses may be better in Asian populations than the Caucasian populations^37^. The sequences of symptom onset observed in patients with NMOSD in the current study was evaluated in 12 patients who first had visual symptoms first and then spinal cord symptoms, four patients who had spinal cord symptoms first and then visual symptoms, 4 patients who had only spinal cord symptoms, and one patient who had one attack characterized by thalamic, midbrain and spinal cord involvement. In these four different sequence groups, there were no significant differences in onset age, MRC scores in all limbs, the level of the anti-AQP4 antibody, one-month outcomes or the lengths of lesions in the spinal cord (all p > 0.05).

### Limitations

Several limitations of the current study should be addressed. First, in this retrospective study, we collected cases over a 16-year sampling period, and it must be acknowledged that MRI protocols and quality could have changed over this time. Some MRI sequences, such as diffusion-weighted imaging of the spine, are technically challenging to acquire and have only recently become available in a clinical setting, in the current study; we used only T1- and T2-weighted images that were reviewed by a single neuroradiologist to minimize discrepancies in imaging data. Second, we focused only on acute noncompressive myelopathies resulting from SCI or NMOSD; thus, other aetiologies, such as vascular malformation (e.g., arteriovenous malformations or fistulas), infection, autoimmune processes and spinal cord lesions related to myelin oligodendrocyte glycoprotein (MOG) antibodies, were not included^13,38^. The temporal profiles of the initial symptoms reported between myelopathies related to ischemic disease and those associated with vascular malformation could be different^13^. We also did not determine the clinical significance of the current MRI features in the diseases mentioned above. A new classification system that could be used in imaging features in acute myelopathy to help clinicians differentiate and predict the outcomes of various aetiologies of acute noncompressive myelopathy is needed. Third, patients with either SCI or NMOSD are relatively rare, and the sample size obtained in this single-institute study was therefore not large enough. The small sample size is likely responsible for the failure to identify relationships between some of the MRI parameters and clinical features. A prospective, multi-centre study is warranted to validate our findings.

## Conclusion

Between the patients with NMOSD and those with SCI evaluated in this study, we identified significant differences in demographic factors, the temporal profiles of symptom onset and short-term outcomes. Some MRI features could help clinicians to make correct differential diagnoses between SCI and NMOSD. Vertebral body infarction signs were significantly associated with aortic pathology. In contrast, long segments of SCI and both ASA and unilateral PSA infarctions were associated with artery dissections. Although prognoses were not previously found to be associated with MRI features, in this study, we show that the owl’s eyes sign is associated with low initial MRC scores, and low MRC scores are associated with poor short-term outcomes at one month; these findings improve the ability of clinicians to make correct differential diagnoses and prognostic predictions between SCI and NMOSD.

### Data sharing statement

Additional clinical data are available from laboratory studies. Please contact Long-Sun Ro (E-mail: cgrols@adm.cgmh.org.tw) if this information is of interest.
